# LPS Induces Active HMGB1 Release From Hepatocytes Into Exosomes Through the Coordinated Activities of TLR4 and Caspase-11/GSDMD Signaling

**DOI:** 10.3389/fimmu.2020.00229

**Published:** 2020-04-03

**Authors:** Wenbo Li, Meihong Deng, Patricia A. Loughran, Muqing Yang, Minjie Lin, Chenxuan Yang, Wentao Gao, Shuqing Jin, Shilai Li, Jingjing Cai, Ben Lu, Timothy R. Billiar, Melanie J. Scott

**Affiliations:** ^1^Department of Burn and Plastic Surgery, The Second Xiangya Hospital of Central South University, Changsha, China; ^2^Department of Surgery, University of Pittsburgh, Pittsburgh, PA, United States; ^3^Center for Biologic Imaging, University of Pittsburgh, Pittsburgh, PA, United States; ^4^Department of Surgery, Tenth People's Hospital of Tongji University, Shanghai, China; ^5^The Second Xiangya Hospital of Central South University, Clinical Skills Training Center, Changsha, China; ^6^School of Medicine, Tsinghua University, Beijing, China; ^7^Department of Anesthesiology, Shanghai East Hospital, Tongji University, Shanghai, China; ^8^Department of Emergency, The First Affiliated Hospital of Guangxi Medical University, Nanning, China; ^9^Department of Cardiology, The Third Xiangya Hospital, Central South University, Changsha, China; ^10^Department of Hematopathology, The Third Xiangya Hospital, Central South University, Changsha, China; ^11^Pittsburgh Liver Research Center, University of Pittsburgh, Pittsburgh, PA, United States

**Keywords:** gasdermin D (GsdmD), endotoxemia, caspase-11, extracellular vesicles, calcium, innate immunity

## Abstract

High-mobility group box-1 (HMGB1), a ubiquitous nuclear protein, acts as a late mediator of lethality when released extracellularly during sepsis. The major source of circulating HMGB1 in sepsis is hepatocytes. However, the mechanism of HMGB1 release of hepatocytes during sepsis is not very clear. We have previously shown that bacterial endotoxin [lipopolysaccharide (LPS)] sensing pathways, including Toll-like receptor (TLR)4 and caspase-11, regulate hepatocyte HMGB1 release in response to LPS. Here, we report the novel function of caspase-11 and gasdermin D (GsdmD) in LPS-induced active HMGB1 released from hepatocytes. HMGB1 release during endotoxemia was caspase-11/GsdmD dependent via an active way *in vivo* and *in vitro*. Caspase-11/GsdmD was responsible for HMGB1 translocation from nucleus to the cytoplasm via calcium changing-induced phosphorylation of calcium-calmodulin kinase kinase (camkk)β during endotoxemia. Cleaved GsdmD accumulated on the endoplasmic reticulum, suggesting this may lead to calcium leak and intracellular calcium increase. Furthermore, we investigated that exosome was an important pathway for HMGB1 release from hepatocytes; this process was dependent on TLR4, independent of caspase-11 and GsdmD *in vivo* and *in vitro*. These findings provide a novel mechanism that TLR4 signaling results in an increase in caspase-11 expression, as well as increased exosome release, while caspase-11/GsdmD activation/cleavage leads to accumulation of HMGB1 in the cytoplasm through a process associated with the release of calcium from the endoplasmic reticulum and camkkβ activation.

## Introduction

Sepsis is a dysregulated inflammatory and metabolic state associated with infection. This dysregulated state is associated with multi-organ dysfunction and high mortality (1). Endotoxin [lipopolysaccharide (LPS)], a constituent of Gram-negative bacteria, stimulates immune and non-immune cells to release excessive levels of inflammatory mediators (e.g., cytokines), which can precipitate tissue injury and lethal shock. However, blocking single cytokines early in the course of sepsis has not improved outcomes during clinical trials (2). This led to the search for late mediators of lethality in sepsis, and this search yielded high-mobility group box-1 (HMGB1), a nuclear protein that is released by the liver during sepsis that can drive pyroptosis, immune dysfunction, and lethality in sepsis models (3, 4).

Our findings (5) and the findings of others (6) established that active release of HMGB1 by hepatocytes is the dominant source of systemic levels of HMGB1 during endotoxemia and sepsis. HMGB1 contributes to lethality in sepsis by delivering extracellular LPS to cytosolic caspase-11 in macrophages and endothelial cells (5). This, in turn, leads to macrophage and endothelial cell pyroptosis that then propagates the systemic inflammatory response and immune dysfunction (7–9). Caspase-11 (caspase 4 and 5 in humans) belongs to the family of inflammatory caspases and is also referred to as the non-canonical inflammasome. The binding of cytosolic LPS to the caspase activation and recruitment domain (CARD) of caspase-11 leads to its oligomerization/activation (8). Active caspase-11 promotes caspase-1 activation, and both caspases cleave gasdermin D (GsdmD) (10, 11). The N-terminal fragment of GsdmD forms 10- to 14-nm pores in artificial or natural phospholipid mixtures (12, 13).

The intracellular steps that lead to the active release of HMGB1 by hepatocytes in response to LPS are unknown. Interestingly, this release is known to involve Toll-like receptor (TLR)4-mediated LPS uptake by hepatocytes and is caspase-11 dependent (5, 14). While TLR4 is required for the upregulation of caspase-11 in hepatocytes exposed to LPS, how these two LPS sensing pathways then regulate the release of HMGB1 is not known. Here, we show that hepatocytes mobilize HMGB1 from the nucleus to the cytosol through a process that requires caspase-11-dependent GsdmD cleavage, increases in intracellular calcium, and calcium-calmodulin kinase kinase (camkk)β activation. We provide evidence that a cleavage fragment of GsdmD inserts into the endoplasmic reticulum (ER) membrane and may initiate calcium-dependent signaling. Extracellular release of HMGB1 takes place via exosomes, and this requires receptor-specific roles for TLR4 and caspase-11/GsdmD. These findings illuminate a novel pathway for the active release of HMGB1 from hepatocytes that is relevant to sepsis lethality.

## Experimental Procedures

### Exosome Isolation and Quantification

Exosome isolation from hepatocyte culture media was performed as described previously (15). Briefly, cell culture media was centrifuged at 500 × g for 10 min, 16,500 × g for 20 min, followed by filtration through a 0.2-μm filter (Life Sciences). Exosomes were pelleted at 120,000 g for 70 min with Type 70.1 Ti rotor (Beckman). The exosomes were further washed once with phosphate buffered saline (PBS) and centrifuged at 120,000 g for 70 min, then resuspended in a small volume of PBS for NanoSight™ assessment or in lysis buffer (1:10 dilution, Cell Signaling Technology, #9803) for Western blot. Plasma exosomes were isolated using the total exosome isolation kit (Invitrogen) according to manufacturer's instructions. The pellet was resuspended in sample dilution buffer for ELISA or lysis buffer for Western blot.

### Animal Model

Male C57BL/6J wild-type (WT) mice were purchased from Jackson Laboratory. GsdmD knockout (KO) mice were obtained from Dr. Vishva Dixit (Genetech). TLR-4 KO (16), caspase-11 KO (5), and GsdmD KO (17) mice on C57BL/6 background were bred in Dr. Billiar's lab. We also generated mice with selective Hmgb1 deletion (5) in either myeloid cells (HMGB1^f/f^ Lyz2-cre^+^) or hepatocytes (HMGB1^f/f^ Alb-cre^+^). All animals were housed or bred in the specific pathogen-free animal facility at the University of Pittsburgh School of Medicine and were kept under a 12-h dark/light cycle, fed standard chow *ad libitum*.

Mice were intraperitoneally (i.p.) injected with 5 mg/kg LPS for the time indicated in the experiments. Knockdown of Rab27a *in vivo* was performed as previously (18). Briefly, 1 × 10^9^ plaque forming unit (PFU) adenoviruses Rab27a shRNA (Vector Biolabs, Malvern, PA) were injected into the penile vein of mice anesthetized by isoflurane. Two days after virus injection, mice were injected i.p. with LPS or saline. For exosome release inhibition *in vivo*, GW4869 dissolved in dimethyl sulfoxide (DMSO) (0.005%) was pre-injected into the penile vein at one dose of 2.5 mg/kg 1 h before LPS treatment.

### Isolation and Culture of Hepatocytes

Cells were isolated from mice by an *in situ* collagenase (type VI; Sigma) perfusion technique, modified as described previously (19). Cell viability was typical >95% by trypan blue exclusion. Hepatocytes (4 × 10^5^ cells/plate for six-well plates, 5 × 10^6^ cells/plate for 10-cm plates) were plated on gelatin-coated culture plates in Williams medium E with 10% calf serum, 15 mM 4-(2-hydroxyethyl)-1-piperazineethanesulfonic acid (HEPES), 10^−6^ M insulin, 2 mM L-glutamine, 100 U/ml penicillin, and streptomycin. Cells were allowed to attach to plates for at least 4 h before treatment.

### Cell Treatment

Primary hepatocytes were treated with or without 1 μg/ml LPS in serum-free liver media (15 mM HEPES, 10^−6^ M insulin, 2 mM L-glutamine, 100 U/ml penicillin, and streptomycin) for 24 h. Culture media from two 10-cm plates for each group were harvested for exosome isolation. Proteins in the supernatant were extracted using methanol/chloroform. Total lysates were prepared using lysis buffer (1:10, Cell Signaling Technology). GW4869 and spiroepoxide were prepared as previously described (20). For exosome inhibition *in vitro*, GW4869 or spiroepoxide was added 2 h before LPS treatment (1 μg/ml LPS for 4 or 8 h). For knockdown of caspase-11 or GsdmD, 300 ng siRNA was diluted in 400 μl serum-free medium with 12 μl HiperFect™ transfection reagent (Qiagen) and mixed by vortexing. The mixture was incubated for 10 min at room temperature, added dropwise to the cells in 2-ml medium with 10% fetal bovine serum (FBS) (final siRNA concentration was 10 nM), swirled, and cultured the cells for 48 h.

### Isolation of Nuclear/Cytoplasmic Protein and Endoplasmic Reticulum

Nuclear and cytoplasmic proteins were prepared using NE-PER™ Nuclear and Cytoplasmic Extraction Reagents (Thermo Fisher) according to the manufacturer's instructions. ER was prepared using an ER isolation kit (Sigma) according to the manufacturer's instructions. Nuclear/cytoplasmic or ER proteins were quantified using Pierce^TM^ BCA protein assay kit (Thermo Fisher).

### Immunofluorescent Staining on Primary Hepatocytes and Tissue Sections

Primary hepatocytes cultured on coverslips were treated as described and then fixed with 2% (w/v) paraformaldehyde in PBS for 15 min. Residual paraformaldehyde was removed by multiple washes with PBS, and cells were permeabilized with 0.1% Triton X-100 in PBS for 15 min at room temperature, washed with PBS and PBB (0.5% BSA in PBS) and blocked for 1 h with 20% normal goat serum (NGS, Sigma) in PBS. Then samples were incubated with the specific primary antibody for HMGB1 (Abcam, 1:200) in 1% BSA for 1 h, washed, and incubated with secondary antibody (goat anti-rabbit-Cy3, Jackson ImmunoResearch, 1:1,000). Additional *in vitro* experiments were performed with primary hepatocytes cultured on coverslips that were treated with Zombie Red™ viability dye (1:1,000, Biolegend) at room temperature in the dark for 30 min. All immunofluroescent staining sets involved staining the nuclei with Hoechst (1 mg/100 ml; Sigma) was applied at room temperature for 30 s followed by a single rinse of PBS to remove excess dye. *In vitro* samples cultured to collagen coated coverslips were adhered on the cell surface side of the coverslip to slides using Gelvatol [23 g of poly(vinyl alcohol 2000), 50 ml of glycerol, 0.1% sodium azide to 100 ml of PBS].

Liver tissue removed after perfusion with cold PBS and 2% paraformaldehyde was incubated for an additional 2 h to complete tissue fixation and then incubated for 24 h in 30% sucrose, followed by cryopreservation in liquid nitrogen cooled 2-methylbutane. Tissue sections of 6 μm were permeabilized with 0.3% Triton X-100 for 20 min, followed by staining according to the manufacturer's protocol of the *in-situ* Cell Death Detection Kit-TMR red (Roche). Samples were washed with PBS prior to being coverslipped using Gelvatol.

Regardless of the source of samples, all imaging conditions were maintained at identical settings with original gating performed using the primary delete control (no primary antibody). Large area images in X and Y were taken at a magnification of 20× with a two-fold digital zoom for the equivalent of nine fields/section with a Nikon A1 confocal microscope (purchased with 1S10OD019973-01 awarded to Dr. Simon C. Watkins). Quantification was performed in a blinded fashion using NIS Elements Software (Nikon). In brief, the Nikon NIS elements quantification software measure amount of cell death (either TMR or Zombie) fluorophore colocalized with the nuclear Hoechst fluorescences. The amount of HMGB1 content was measured for total HMGB1 fluorescences, as well as the amount of HMGB1 that colocalized with the nuclear content to enable the reporting of nuclear HMGB1, and cytosolic HMGB1 was analyzed as the amount of HMGB1 that did not colocalize with the nuclear HMGB1 content.

### Liver Damage Assessment

Mouse plasma was used for alanine aminotransferase (ALT) test. ALT levels were measured using the DRI-CHEM 4000 Chemistry Analyzer System (Heska). The ALT values were expressed as international units per liter.

### Intracellular Ca^2+^ Measurement

Cells were plated on a 96-well black clear bottom plate. After LPS treatment, cells were washed and loaded with the ratiometric Ca^2+^ indicator Fura-2/AM in calcium-free Hank's balanced salt solution (HBSS) [at 37°C, 5% carbon dioxide (CO_2_)] for 30 min, washed, and incubated for an additional 30 min prior to testing. Excitation was carried out at 340 and 380 nm, and emissions were collected at 510 ± 10 nm using BioTek SynergyMx multi-format microplate readers.

### Exosome NanoSight™ Analysis (Nano Tracking Analysis)

Exosome samples were analyzed as previously described (21). Briefly, exosomes isolated from 100 μl plasma were resuspended in 100 μl PBS and diluted 1:10,000 in particle-free water (W4502, Sigma). Exosomes isolated from 10^7^ cells were resuspended in 50 μl PBS and diluted 1:10,000 in particle-free water. After vortexing, the diluted samples were injected into the NTA LM-10 system continuously using a syringe pump. Particles were acquired by the machine, and data were analyzed with NTA particle analysis software.

### ELISA Assay

HMGB1 ELISA Kit (IBL, Hamburg, Germany) was used to detect plasma HMGB1 levels according to the manufacturer's instructions. CD81 ELISA Kit (Cusabio, Wuhan, China) was used to detect plasma exosome samples according to the manufacturer's instructions.

### Western Blot

Antibodies for Western blot analysis were as follows: anti-HMGB1 (1:1,000, Abcam), anti-caspase-11 (1:500, Sigma), anti-TSG101 (1:500, Novus), anti-CD81 (1:500, Novus), anti-Rab27a (1:1,000, Abcam), anti-Rab27b (1:1,000, Abcam), anti-beta actin (1:5,000, Abcam), anti-glyceraldehyde 3-phosphate dehydrogenase (GAPDH) (1:5,000, Bio-Rad), anti-tubulin (1:5,000, Bio-Rad), anti-specificity protein 1 (SP1) (1:500, Santa Cruz), anti-phospho-camkk2 (1:1,000, Cell Signaling), anti-camkk2 (1:1,000, Novus), anti-calnexin (1:1,000, Novus), anti-ERp72 antibody (1:1,000, Cell Signaling). Secondary antibodies (1:10,000) were from Thermo Fisher Scientific. The procedure of Western blot analysis was as previously described (22). For *in vitro* experiments, hepatocytes were washed with PBS at the endpoint of experiments, collected in lysis buffer (Cell Signaling Technology) with phenylmethylsulfonyl fluoride (PMSF) and protease inhibitors, and centrifuged at 16,000*g* for 10 min, and supernatant was collected for Western blotting. For *in vivo* experiments, frozen liver (ischemic lobe) was homogenized in lysis buffer and centrifuged at 16,000*g* for 10 min, and supernatant was collected. Protein concentrations of the supernatants were determined with the bicinchoninic acid (BCA) protein assay kit (Thermo Fisher Scientific). Sodium dodecyl sulfate (SDS) loading buffer was then added to the samples. Denatured protein samples were analyzed by 10% or 15% SDS–polyacrylamide gel electrophoresis and then transferred onto a polyvinylidene difluoride membrane at 250 mA for 2 h. The membrane was blocked in 5% milk (Bio-Rad) in Tris-buffered saline (TBS) for 1 h and then incubated overnight with primary antibody in 1% milk in TBS overnight. Membranes were washed three times in TBS-Tween (TBS-T) for 10 min, incubated with horseradish peroxidase-conjugated secondary antibody for 1 h, and then washed three times for 10 min in TBS-T before being developed for chemiluminescence (Bio-Rad). Densitometry analysis was performed using the ImageJ Gel Analysis tool. GAPDH, β-actin, and tubulin are used as loading controls.

### Statistical Analysis

All data were analyzed using GraphPad Prism software (version 6.0). For *in vivo* and *in vitro* experiments, numerical measures will be compared using Student's *t*-test and were used for comparison between two groups or one-way ANOVA followed by *post hoc* Bonferroni test for multiple comparisons. If the data do not satisfy the assumptions necessary for this analysis, variables will be transformed, or a non-parametric alternative will be used. For the test above, there will be enough cultures/mice to attain a power of at least 80% at a significance level of 0.05. The required effect sizes for each analysis were estimated using the results from the preliminary studies. Based on these numbers, it was confirmed that the planned sample sizes are sufficient in attaining the desired power. Further, for statistical analysis of our *in vitro* studies, data will be expressed as mean ± SEM of three independent experiments performed in triplicate. A *p* < 0.05 was considered statistically significant for all experiments. All values are presented as the mean ± SEM.

## Results

### Lipopolysaccharide-Induced High-Mobility Group Box-1 Release From Hepatocytes Is Caspase-11 and Gasdermin D Dependent

**Figure 1:**
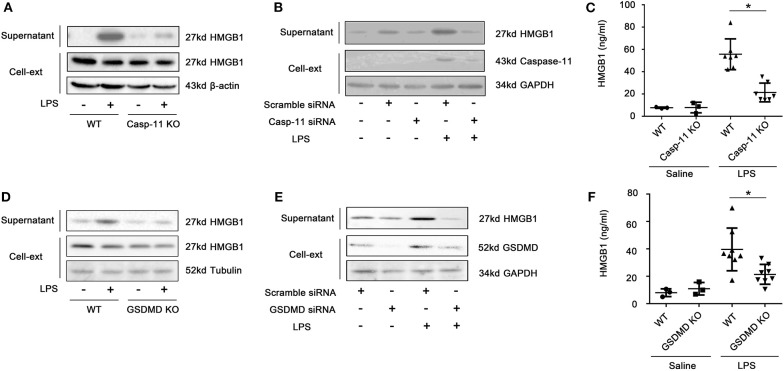
Lipopolysaccharide (LPS)-induced high-mobility group box-1 (HMGB1) release from hepatocytes is caspase-11 and gasdermin D (GsdmD) dependent. Immunoblots for HMGB1, β-actin, glyceraldehyde 3-phosphate dehydrogenase (GAPDH), or tubulin in the supernatant and cell lysates (Cell-Ext) in **(A)** wild-type (WT) and caspase-11^−/−^ [caspase-11 knockout (KO)] and **(D)** WT and GsdmD^−/−^ (GsdmD KO) hepatocytes at 24 h after LPS (1 μg/ml). **(B,E)** Hepatocytes pretreated with siRNA to knock down caspase-11 or GsdmD prior to LPS treatment for 24 h as above. **(C,F)** Plasma HMGB1 levels in WT, caspase-11^−/−^, or GsdmD^−/−^ mice at 4 h after intraperitoneal injection with LPS (5 mg/kg). Each point represents one mouse. *P < 0.05. *n* = 3.

**Figure 2:**
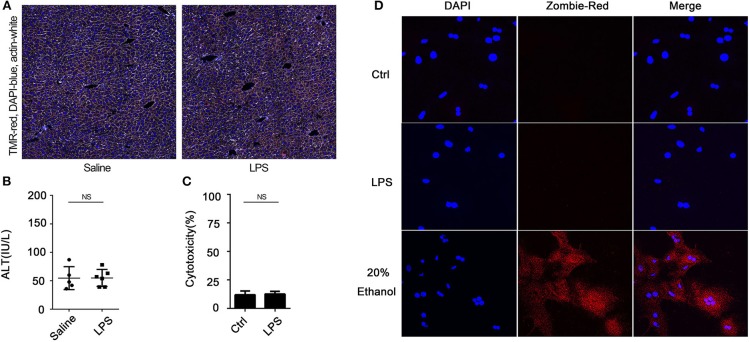
Lipopolysaccharide (LPS) does not induce hepatocyte death *in vitro* or *in vivo*. **(A)** Immunofluorescence of liver from wild-type (WT) mice at 4 h after intraperitoneal injection with LPS (5 mg/kg). TMR = red; 4′,6-diamidino-2-phenylindole (DAPI) = blue; actin = white. **(B)** Plasma alanine aminotransferase (ALT) level in WT mice at 4 h after intraperitoneally injection with LPS (5 mg/kg). Each point represents one mouse. **(C)** WT hepatocytes were treated with LPS (1 μg/ml) for 24 h. Cytotoxicity was measured by using lactate dehydrogenase (LDH) release in the culture media. Data are expressed as mean ± SEM. **(D)** Immunofluorescence of Zombie-red staining (cell death) of WT hepatocytes 24 h after treatment with LPS (1 μg/ml). DAPI = blue. NS, no significant difference. *n* = 3.

We have previously demonstrated that hepatocytes release HMGB1 in sepsis and that this requires caspase-11 and GsdmD (5). Here, we confirmed that hepatocytes are the dominant source of the increases in circulating HMGB1 during endotoxemia. As shown in Supplemental Figure 1, cell-specific deletion of HMGB1 in hepatocytes, but not myeloid cells, prevented the rise in plasma HMGB1 observed in mice after LPS injection. As expected, LPS treatment *in vivo* led to an increase in liver levels of caspase-11 by 4 h that further increased at 8 h. The levels of caspase-11 at 24 h decreased to a similar level as at 4 h (Supplemental Figure 2). We confirmed that LPS treatment of cultured hepatocytes led to a caspase-11-dependent cleavage of GsdmD (Supplemental Figure 3). Deletion or knockdown of caspase-11 or GsdmD prevented LPS-induced HMGB1 release by cultured hepatocytes (Figures 1A,B,D,E; Supplemental Figure 6), while deletion of caspase-11 or GsdmD prevented the circulating rise in HMGB1 following LPS treatment *in vivo* (Figures 1C,F). The increase in extracellular HMGB1 induced by LPS treatment *in vitro* and *in vivo* was not due to cell death (Figure 2). LPS treatment *in vivo* failed to increase terminal deoxynucleotidyl transferase dUTP nick end labeling (TUNEL)-positive cells in the liver or ALT levels in the plasma. Furthermore, LPS, at a concentration shown to induce HMGB1 release from hepatocytes *in vitro*, did not increase lactate dehydrogenase (LDH) release or Zombie-red uptake in cultured WT hepatocytes. Thus, hepatocytes actively release HMGB1 after exposure to LPS via a process that requires caspase-11 and GsdmD.

### Caspase-11 and Gasdermin D Are Required for High-Mobility Group Box-1 Translocation From the Nucleus to the Cytoplasm

**Figure 3:**
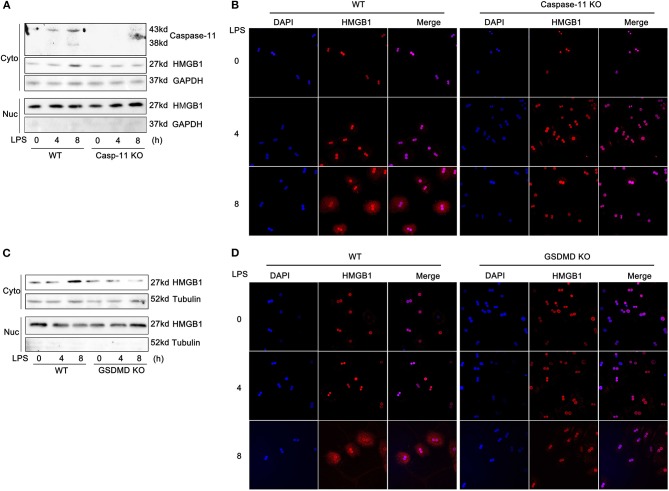
Caspase-11 and gasdermin D (GsdmD) are required for high-mobility group box-1 (HMGB1) translocation to the cytosol in response to lipopolysaccharide (LPS). **(A,C)** Immunoblots for HMGB1, caspase-11, glyceraldehyde 3-phosphate dehydrogenase (GAPDH), or tubulin in the cytoplasmic (Cyto) and nuclear (Nuc) lysates from wild-type (WT), caspase-11^−/−^ [Casp-11 knockout (KO)], or GsdmD^−/−^ (GsdmD KO) hepatocytes treated with LPS (1 μg/ml) for the time indicated. **(B,D)** Immunofluorescence of WT, caspase-11^−/−^, or GsdmD^−/−^ hepatocytes treated with LPS (1 μg/ml) for the indicated time. HMGB1 = red; 4′,6-diamidino-2-phenylindole (DAPI) = blue; colocalization = magenta. *n* = 3.

The active secretion of HMGB1 from cells requires at least two steps. First, HMGB1 accumulates in the cytoplasm, instead of the nucleus, and second, HMGB1, a leaderless protein, is released into the extracellular space (23). Therefore, we next assessed whether the nucleo-cytoplasmic translocation of HMGB1 in hepatocytes following LPS treatment required caspase-11 and/or GsdmD. As shown in Figure 3 and Supplemental Figure 7, LPS treatment led to an increase in cytosolic HMGB1 levels by 8 h. Whereas, deletion of caspase-11 or GsdmD had no impact on baseline levels of nuclear HMGB1 in hepatocytes, deletion of either gene prevented the accumulation of HMGB1 in the cytoplasm induced by LPS exposure. Specificity protein 1 (SP1) and GAPDH/tubulin were used to verify the compartment specificity of the proteins isolated from the nucleus and cytoplasm (**Figure 5B**; Supplemental Figure 9). These data establish that caspase-11 and GsdmD are involved in the nucleo-cytoplasmic translocation of HMGB1 that occurs after exposure of hepatocytes to extracellular LPS.

### Caspase-11 and Gasdermin D Are Required for Lipopolysaccharide-Induced Phosphorylation of Camkkβ

**Figure 4:**
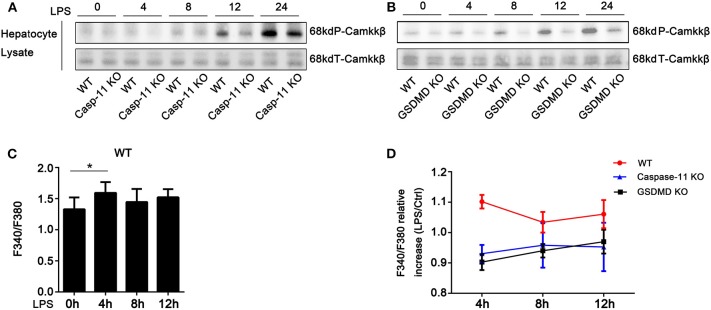
Caspase-11 and gasdermin D (GsdmD) inhibited the phosphorylation of calcium-calmodulin kinase kinase (camkk)β. **(A,B)** Immunoblots for phospho-camkkβ (P-camkkβ) and total-camkkβ (T-camkkβ) in whole cell lysates from wild-type (WT), caspase-11^−/−^ [Casp-11 knockout (KO)], or GsdmD^−/−^ (GsdmD KO) hepatocytes were treated with lipopolysaccharide (LPS) (1 μg/ml) for the indicated times. **(C)** Intracellular Ca^2+^ measured by fluorescence intensity of Fura-2AM (F340/F380) in WT hepatocytes treated with or without LPS (1 μg/ml) for the indicated time. Data are expressed as mean ± SEM. **(D)** Intracellular Ca^2+^ measured by fluorescence intensity of Fura-2AM (F340/F380) in WT, caspase-11^−/−^, or GsdmD^−/−^ hepatocytes treated with or without LPS (1 μg/ml) at indicated times. Data are expressed as relative levels compared with baseline controls and as mean ± SEM. ^*^P < 0.05. *n* = 3.

We have previously shown that hypoxia-induced HMGB1 release by hepatocytes requires camkkβ (24). Camkkβ belongs to the serine/threonine-specific protein kinase family and to the Ca^2+^/calmodulin-dependent protein kinase subfamily. Camkkβ catalyzes the phosphorylation of threonine residues located in the activation loop of the CaMKI and CaMKIV, enhancing their kinase activity. Therefore, we tested whether inhibition of camkkβ would reduce HMGB1 release from hepatocytes exposed to LPS. STO-609, a specific camkk inhibitor, blocked LPS-induced HMGB1 release (Supplemental Figure 4A). Interestingly, previous reports suggest that camkkβ (25) and its downstream targets, CaMKI (26, 27) and CaMKIV (28), may regulate HMGB1 nucleo-cytoplasmic translocation. Therefore, we investigated whether caspase-11 or GsdmD were required for camkkβ pathway activation. As shown in Figure 4 and Supplemental Figure 8, deletion of either caspase-11 or GsdmD reduced the phosphorylation of camkkβ in hepatocytes exposed to LPS (Figures 4A,B). Consistent with this result, we also found that intracellular free calcium was increased following LPS treatment in hepatocytes, and this required caspase-11 and GsdmD (Figures 4C,D). Furthermore, A23187, an ionophore that increases intracellular calcium levels, promoted HMGB1 release in response to LPS in caspase-11^−/−^ and GsdmD^−/−^ hepatocytes to a level similar to that seen in WT hepatocytes (Supplemental Figure 4B). Taken together, these data show that the Ca^2+^ signaling regulates LPS-induced HMGB1 release in hepatocytes, and this signaling pathway requires both caspase-11 and GsdmD.

### Lipopolysaccharide Triggers Gasdermin D Association With the Endoplasmic Reticulum

**Figure 5:**
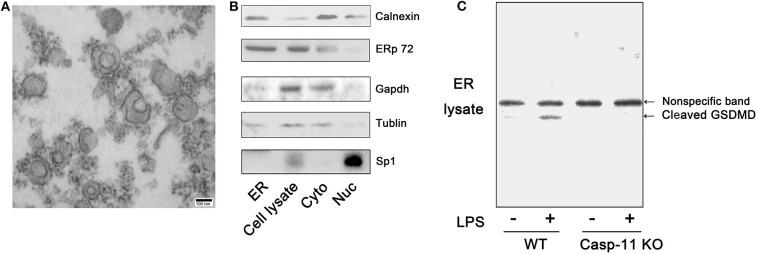
Gasdermin D (GsdmD) is recruited to the endoplasmic reticulum (ER). **(A)** Morphological structure of isolated mouse ER as seen on standard transmission electron microscopy (TEM) (100,000 × magnification; scale bar, 100 nm). **(B)** Immunoblots for calnexin, ERp72, glyceraldehyde 3-phosphate dehydrogenase (GAPDH), tubulin, and specificity protein 1 (SP1) in isolated ER, whole cell (Cell lysate), cytoplasm (Cyto), and nucleus (Nuc) of wild-type (WT) hepatocytes. **(C)** Immunoblots for GsdmD in ER (ER lysate) isolated from WT and caspase-11^−/−^ [Casp-11 knockout (KO)] hepatocytes after treatment with lipopolysaccharide (LPS) (1 μg/ml) for 8 h. *n* = 3.

Next, we investigated how GsdmD regulates intracellular calcium transients. Calcium storage is one of the functions commonly attributed to the ER in non-muscle cells (29). The N-terminal cleavage fragment of GsdmD can form pores in phospholipid membranes (13). Therefore, we sought evidence for GsdmD association with the ER in LPS-treated hepatocytes. The purity of the isolated ER was confirmed using electron microscopy and the ER protein markers, calnexin and ERp72 (Figures 5A,B). Western blot analysis demonstrated the presence of a GsdmD cleavage fragment in the ER lysate from LPS-treated WT hepatocytes but not caspase-11^−/−^ hepatocytes (Figure 5C). These findings raise the possibility that caspase-11 cleaves GsdmD, and the cleavage fragment then inserts in the ER membrane to release calcium into the cytoplasm.

### High-Mobility Group Box-1 Is Released From Hepatocytes in Exosomes

**Figure 6:**
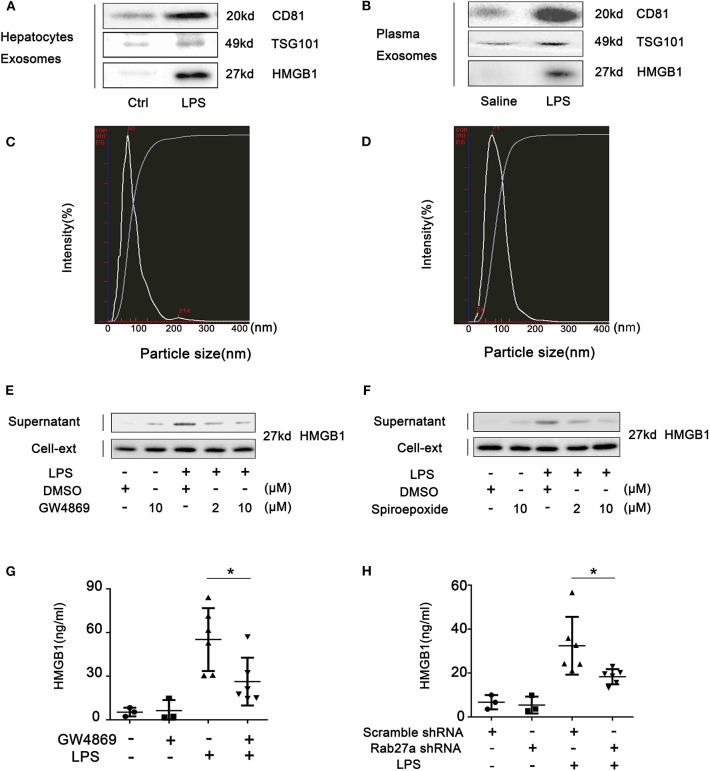
Hepatocytes release high-mobility group box-1 (HMGB1) in exosomes. Immunoblots for CD81, TSG101, and HMGB1 in exosomes isolated from **(A)** cell culture media from wild-type (WT) hepatocytes treated with lipopolysaccharide (LPS) (1 μg/ml) for 24 h and **(B)** plasma from WT mice intraperitoneally injected with LPS (5 mg/kg) for 4 h. NanoSight™ analysis of exosomes isolated from **(C)** cell culture media and **(D)** mouse plasma. **(E,F)** Immunoblots for HMGB1 in the supernatant and cell lysates of hepatocytes treated with GW4869 or spiroepoxide for 2 h, then challenged with LPS (1 μg/ml) for 24 h. **(G)** Plasma HMGB1 level in WT mice injected intravenously with GW4869 (2.5 mg/kg) for 1 h prior to intraperitoneal injection of LPS (5 mg/kg) for 4 h. **(H)** Plasma HMGB1 level in WT mice pretreated for 48 h with scrambled (control) or Rab27a-targeted shRNA via intravenous injection followed by intraperitoneal injection with LPS (5 mg/kg) for 4 h. Each point represents one mouse. *P < 0.05. *n* = 3.

We have recently provided evidence that HMGB1 is released into the extracellular space inside vesicles (5). To determine if these HMGB1-containing vesicles were exosomes, we confirmed that HMGB1 found in cell supernatant and plasma after LPS challenge was within CD81- and TSG101-positive vesicles (Figures 6A,B; Supplemental Figure 10). To further establish that HMGB1 is released via exosomes, NanoSight™ nanoparticle tracking analysis was used to show that mean size of particles isolated from hepatocyte supernatant *in vitro* and plasma were both in the range consistent for exosomes (40–100 nm) (Figures 6C,D). GW4869 and spiroepoxide, inhibitors of neutral sphingomyelinase associated with exosome release (20), reduced HMGB1 release from hepatocytes in a dose-dependent manner (Figures 6E,F). *In vivo*, GW4869 treatment before LPS challenge significantly suppressed HMGB1 levels in plasma (Figure 6G). Rab27a is required for exosome release (30). An adenovirus-expressing shRNA targeting Rab27a was used to suppress liver Rab27a (Supplemental Figure 5). Knockdown of Rab27a also significantly prevented HMGB1 increases in the plasma of LPS-treated mice (Figure 6H). Combined, these observations support the conclusion that HMGB1 is released into the extracellular space in exosomes.

### High-Mobility Group Box-1 Release in Exosomes Is Toll-Like Receptor 4, Caspase-11, and Gasdermin D Dependent During Endotoxemia

**Figure 7:**
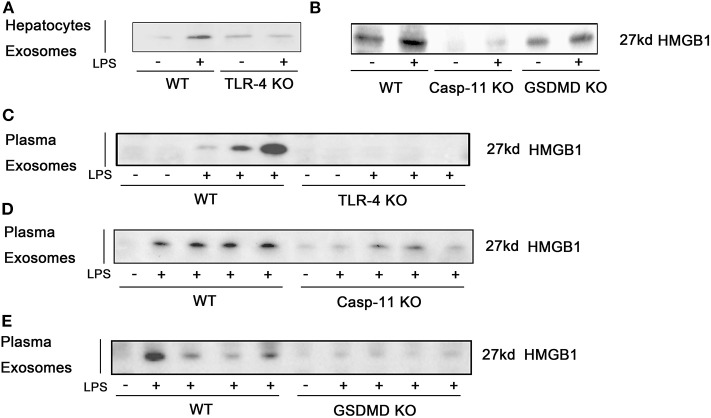
High-mobility group box-1 (HMGB1) release in exosomes is dependent on Toll-like receptor (TLR)4, caspase-11, and gasdermin D (GsdmD). **(A,B)** Immunoblots for HMGB1 in exosomes isolated from cell culture media from wild-type (WT), TLR4^−/−^ [TLR4 knockout (KO)], caspase-11^−/−^ (Casp-11 KO), or GsdmD^−/−^ (GsdmD KO) hepatocytes were treated with lipopolysaccharide (LPS) (1 μg/ml) for 24 h. **(C–E)** Immunoblots for HMGB1 in exosomes isolated from plasma of WT, TLR4^−/−^, caspase-11^−/−^, or GsdmD^−/−^ mice treated with LPS (5 mg/kg) for 4 h. Each lane represents one mouse. *n* = 3.

**Figure 8:**
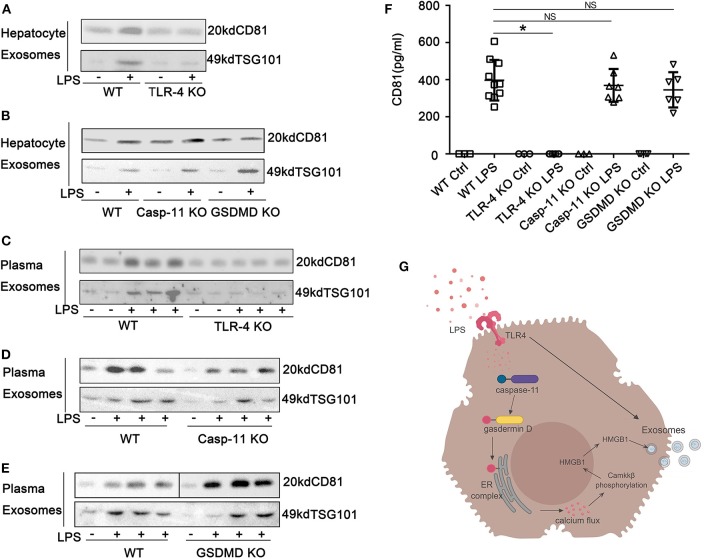
Exosome release from hepatocytes is dependent on Toll-like receptor (TLR)4 but independent of caspase-11 and gasdermin D (GsdmD). **(A,B)** Immunoblots of CD81 and TSG101 in exosomes isolated from cell culture media of wild-type (WT), TLR4^−/−^, caspase-11^−/−^, or GsdmD^−/−^ hepatocytes treated with lipopolysaccharide (LPS) (1 μg/ml) for 24 h. **(C–E)** Immunoblots for CD81 and TSG101 in exosomes isolated from plasma of WT, TLR4^−/−^, caspase-11^−/−^, or GsdmD^−/−^ mice treated with LPS (5 mg/kg) for 4 h. Each lane represents one mouse. **(F)** CD81 levels in exosomes isolated from plasma of WT, TLR4^−/−^, caspase-11^−/−^, or GsdmD^−/−^ mice treated with LPS (5 mg/kg) for 4 h. Each point represents one mouse. **(G)** A proposed model describing TLR4 signaling results in an increase in caspase-11 expression, as well as increased exosome release, while caspase-11/GsdmD activation/cleavage leads to accumulation of high-mobility group box-1 (HMGB1) in the cytoplasm through a process associated with the release of calcium from the endoplasmic reticulum and calcium-calmodulin kinase kinase (camkk)β activation. *P < 0.05. NS, no significant difference. *n* = 3.

We show above that the nucleo-cytoplasmic translocation of HMGB1 and the active extracellular release of HMGB1 in response to LPS exposure both require TLR4 and caspase-11/GsdmD. We hypothesized that the release of HMGB1 into exosomes would depend on the same pathways. Using KO mice, we confirmed that LPS-induced HMGB1 release in exosomes was TLR4, caspase-11, and GsdmD dependent both *in vitro* (Figures 7A,B; Supplemental Figure 11) and *in vivo* (Figures 7C–E). We next asked whether the TLR4 and caspase-11 pathways regulated total exosome release in response to LPS. LPS treatment markedly increased exosome numbers based on the Western blots for CD81 and TSG101. Only the deletion of TLR4 and not the deletion of caspase-11 or GsdmD prevented exosome release into the cell supernatant of cultured hepatocytes or into the plasma of mice after the LPS challenge (Figures 8A–E; Supplemental Figure 8). To further confirm these findings, we used ELISA for quantification of CD81, and the results were consistent with the Western blot analysis (Figure 8F). Thus, TLR4 regulates exosome formation, while caspase-11 and GsdmD are required only for HMGB1 delivery into exosomes.

## Discussion

In this study, we sought to establish the mechanisms involved in the LPS-induced release of HMGB1 into the extracellular space by hepatocytes. This area of investigation is important because active HMGB1 release by hepatocytes has been shown to be critical in the pathogenesis of not only sepsis lethality but also many liver-based diseases (5, 31). Hepatocytes are known to sense the presence of pathogen-associated molecular patterns (PAMPs) in the circulation, and the detection of PAMPs triggers the release of immune regulators such as HMGB1 or chemokines (5, 14, 32). Taken together, our previously published findings (5, 14) combined with our current work demonstrate that hepatocytes utilize surface TLR4 to detect and take up LPS, which occurs concurrently with TLR4-dependent upregulation of caspase-11. Intracellular LPS leads to caspase-11-dependent cleavage of GsdmD, and this promotes increases in free calcium in the cell, camkkβ activation, and relocation of nuclear HMGB1 to the cytoplasm. This is followed by a TLR4- and caspase-11/GsdmD-dependent release of HMGB1 in exosomes. The localization of a cleavage fragment of GsdmD in the ER in LPS-treated cells suggests that the source of free calcium in this signaling cascade may be the ER. These findings introduce a novel mechanism involving the coordinated interaction between the two canonical LPS sensing pathways, surface TLR4 and cytoplasmic caspase-11, for the secretion of HMGB1 into exosomes by hepatocytes. Our observations also raise the possibility that targeting caspase-11 in sepsis could improve outcomes not only by directly blocking pyroptosis in macrophages and endothelial cells but also by suppressing HMGB1 release from the liver.

Our understanding of LPS sensing by immune cells evolved with the discovery that LPS is recognized not only by the cell surface TLR4 receptor complex (33) but also by cytosolic caspase-11 in mice and caspases 4/5 in humans (8). In macrophages and endothelial cells, LPS triggers an inflammatory program aimed at initiating antimicrobial defenses by interacting with cell surface TLR4 (34–36). This inflammatory signaling promotes the upregulation of caspase-11 in the cytosolic compartment of macrophages (37). The delivery of LPS to the cytosol in these cells requires the endocytic uptake of LPS-containing outer membrane vesicles from live Gram-negative bacteria (38), the uptake of live bacteria, and the release of LPS from phagolysosomes, or the uptake of LPS–HMGB1 complexes via cell surface RAGE followed by the release of LPS from lysosomes when HMGB1 destabilizes lysosomal membranes (5). The release of LPS into the cytosol following uptake of bacteria occurs through the actions of interferon-induced GTP-ases (39). The only known consequence of LPS-induced activation of caspase-11 in macrophages or endothelial cells is pyroptosis, which is thought to be a mechanism for the destruction of intracellular microbial niches and the release of local pro-inflammatory molecules [e.g., interleukin (IL)-1α and HMGB1]. Our previous and current work shows that LPS sensing by hepatocytes has a number of characteristics that are unique from macrophages and endothelial cells. Hepatocytes utilize a cell surface TLR4 receptor complex that incorporates CD14 and CD11b/CD18 to uptake LPS into the cell (14). We show here that this response to TLR4 stimulation includes an increase in caspase-11 expression and an increase in exosome numbers released by hepatocytes. In contrast to the cell death seen in macrophages and endothelial cells, caspase-11 activation and GsdmD cleavage in hepatocytes do not lead to cell injury or death but instead mobilize HMGB1 from the nucleus to be released into the exosomes. This coordinated TLR4 and caspase-11/GsdmD interaction represents a novel pathway activated by LPS sensing by hepatocytes. This pathway leads to the massive systemic release of the alarmin/damage-associated molecular pattern (DAMP) and LPS binding protein, HMGB1, from the liver.

Mechanisms for cellular release of HMGB1 fall under two broad categories: passive and active (40, 41). Passive release follows necrosis or programmed cell death, while active release follows a posttranslational modification of nuclear HMGB1. Critical in this process is the acetylation of lysines in the two nuclear localization domains present in HMGB1 (42–44). Although we cannot rule out the possibility that low levels of cell death contribute to the systemic release of HMGB1 during endotoxemia, our results indicate that most of the HMGB1 released by hepatocytes in response to LPS is through an active process that requires both TLR4- and caspase-11-dependent signaling steps. Deletion of TLR4, caspase-11, or the downstream cleavage target of caspase-11, GsdmD, prevents the nucleo-cytoplasmic translocation of HMGB1 induced by LPS. These findings parallel our previous findings where hypoxia-induced HMGB1 release by hepatocytes involves the transfer of acetylated HMGB1 from the nucleus to cytoplasm following the inhibition of nuclear histone deacetylase-1 (HDAC1) and the transfer of histone deacetylase-4 (HDAC4) from the nucleus to the cytoplasm (42). HMGB1 acetylation may also involve an upregulation of histone acetyltransferase in response to Janus kinase/signal transducer and activator of transcription (JAK/STAT) signaling downstream of TLR4 (45, 46). Others have linked JAK/STAT signaling to HMGB1 hyperacetylation (27) and HDAC4 degradation in macrophages (47). We have also shown that hypoxia-induced hepatocyte HMGB1 release requires intracellular calcium signaling through camkkβ and CaMKIV downstream of TLR4 (24), and that CaMK signaling is upstream of HDAC inhibition. Our findings that caspase-11/GsdmD are required for calcium increases in LPS-treated hepatocytes and that the inhibitor of camkkβ blocks LPS-induced HMGB1 release from hepatocytes suggest that caspase-11/GsdmD regulate HMGB1 through calcium signaling, while TLR4 signaling upregulates caspase-11 expression and may regulate acetylation of HMGB1 through JAK/STAT. We speculate that the insertion of the N-terminal fragment of GsdmD into the ER membrane may explain the caspase-11/GsdmD-dependent increase in cytosolic calcium observed in our experiments. This would represent a novel function for cleaved GsdmD but will require further proof that the source of cytosolic calcium is indeed the ER in hepatocytes.

Our data suggest that caspase-11 or GsdmD transiently regulates the calcium flux in hepatocytes at an early time point after LPS treatment, which is prior to the upregulation of caspase-11 levels. Unlike macrophage, there is a measurable amount of caspase-11 in hepatocytes at baseline. Therefore, caspase-11 may not be needed to upregulate in hepatocytes. However, caspase-11 at baseline may be activated at early time points to cause the early (within 4 h) Ca^2+^ increase after GsdmD cleavage and localization to ER. This then activates camkk and HMGB1 translocation. The increase of caspase-11 by later time points may be responsible for other functions, such as packaging and release of exosomes. However, the mechanisms remain elusive.

If the transfer of HMGB1 from the nucleus to the cytoplasm is the first step in the active release of HMGB1 in response to LPS, our findings also support the notion that delivery of cytosolic HMGB1 into exosomes is the second step. Exosomes form in the cytoplasm when cytosolic contents are packaged into multivesicular bodies (48). By blocking factors critical to exosome release including neutral sphingomyelinase or the GTPase Rab27a, we prevented extracellular HMGB1 release in response to LPS, suggesting that HMGB1 is one of the cytosolic proteins that are incorporated as cargo in the exosomes released in response to LPS. Part of the mechanism for exosome release appears to be an increase in overall exosome formation in response to LPS-induced TLR4 signaling, while both TLR4 and caspase-11/GsdmD are required for HMGB1 to accumulate in the cytoplasm. How HMGB1 in the cytoplasm is selected for transfer into exosomes is not known. Furthermore, it is unclear whether the secreted HMGB1 could further act as an internalization signal, thereby mediating the switch-off of the pathway.

In summary, we provide evidence that hepatocytes sense LPS through both TLR4 and caspase-11, and this leads to the active release of HMGB1 in exosomes. As depicted in Figure 8G, each of the LPS-sensing pathways plays unique but interconnected roles in this signaling cascade. An important conceptual advance of this work is that hepatocytes utilize TLR4 and caspase-11 in ways that are distinct from macrophages. The two most striking differences are that TLR4 signaling is critical to the delivery of LPS into hepatocytes, and that the caspase-11/GsdmD pathway does not lead to pyroptosis but instead promotes calcium-dependent signaling for the active release of HMGB1. The disease implications relate to the recently discovered central roles of hepatocyte-derived HMGB1 to sepsis pathogenesis and inflammatory diseases of the liver.

## Data Availability Statement

The datasets generated for this study are available on request to the corresponding author.

## Ethics Statement

This animal study was reviewed and approved by University of Pittsburgh Institutional Animal Care and Use Committee.

## Author Contributions

TB, MS, and BL conceived the project, designed the experiments, analyzed the data, and wrote the paper. WL and MD designed the experiments, performed the experiments, analyzed the data, and wrote the paper. MY, ML, CY, WG, SL, SJ, and JC helped to perform to the experiments. PL performed imaging experiments and edited the manuscript.

### Conflict of Interest

The authors declare that the research was conducted in the absence of any commercial or financial relationships that could be construed as a potential conflict of interest.
