# Supplementary material for: LPS Induces Active HMGB1 Release From Hepatocytes Into Exosomes Through the Coordinated Activities of TLR4 and Caspase-11/GSDMD Signaling
Source: Front Immunol. 2020 Apr 3;11:229. doi: 10.3389/fimmu.2020.00229 (PMC7160675; doi:10.3389/fimmu.2020.00229)
Supplement: Supplementary file 1 [file Data_Sheet_1.PDF]

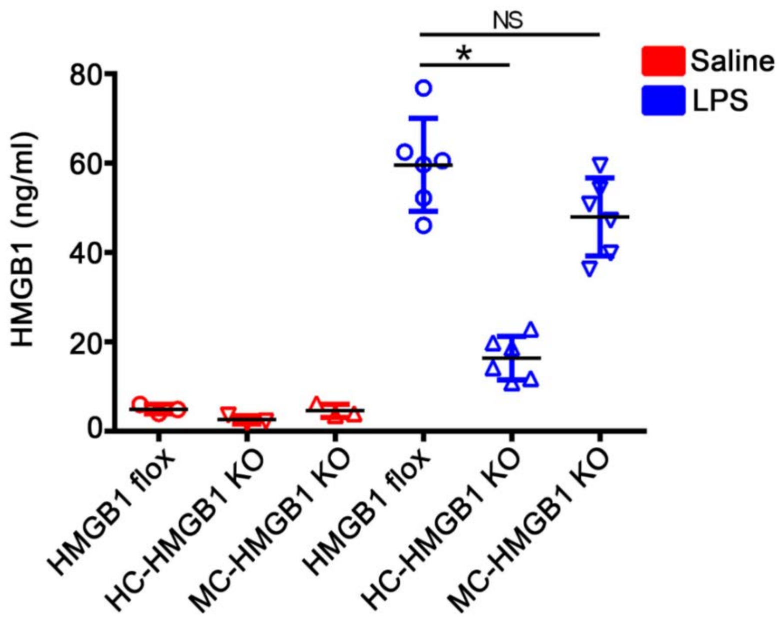

Supplemental Fig 1. Hepatocytes are the dominant source of the increases in circulating HMGB1 during endotoxemia.

HMGB1 flox, hepatocytes specific HMGB1 knockout mice (HC-HMGB1 KO) and myeloid cells specific HMGB1 knockout mice (MC-HMGB1 KO) were intraperitoneally injected with LPS(5mg/kg) for 4h. Plasma HMGB1 levels were tested by Elisa. Each symbol represents one mouse. * $P<0.05$. NS: no significant difference.

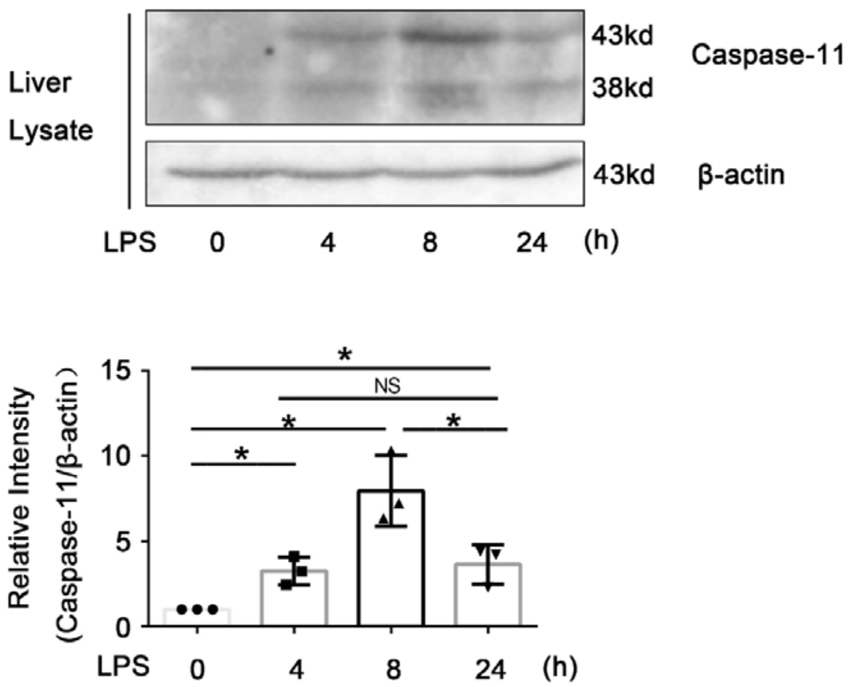

Supplemental Fig 2. LPS treatment in vivo led to an increase in liver levels of caspase-11

WT mice were treated with LPS(5mg/kg) as indicated time point. The Immuno-blots image shows one representative image for caspase-11 and β-actin in the liver tissue lysate. Quantification charts show mean ± SEM of Caspase-11 to β-actin of three individual experiment.*P<0.05. NS: no significant difference.

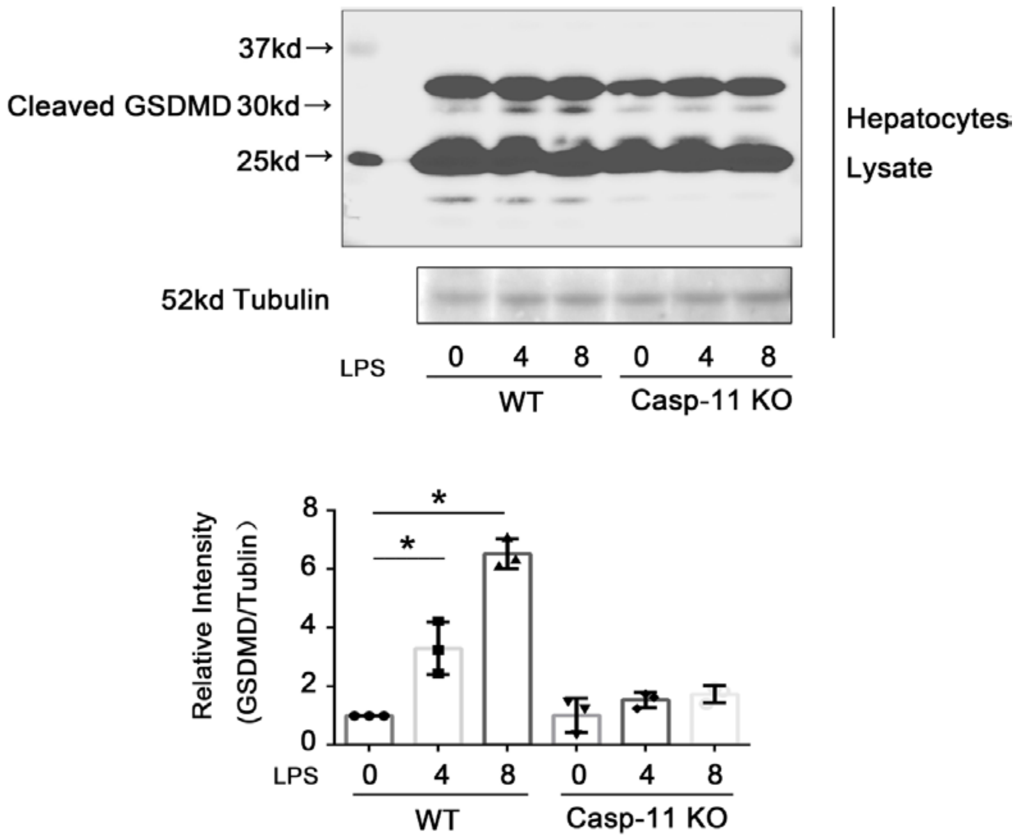

Supplemental Fig 3. LPS treatment of cultured hepatocytes led to a caspase-11 dependent cleavage of GSDMD

WT or caspase-$11^{-/-}$ hepatocytes were treated with LPS(1ug/ml) as indicated time point.

The Immuno-blots image shows one representative image for GSDMD and Tublin in hepatocytes lysate. Quantification charts show mean ± SEM of GSDMD to Tublin of three individual experiments. *$P<0.05$.

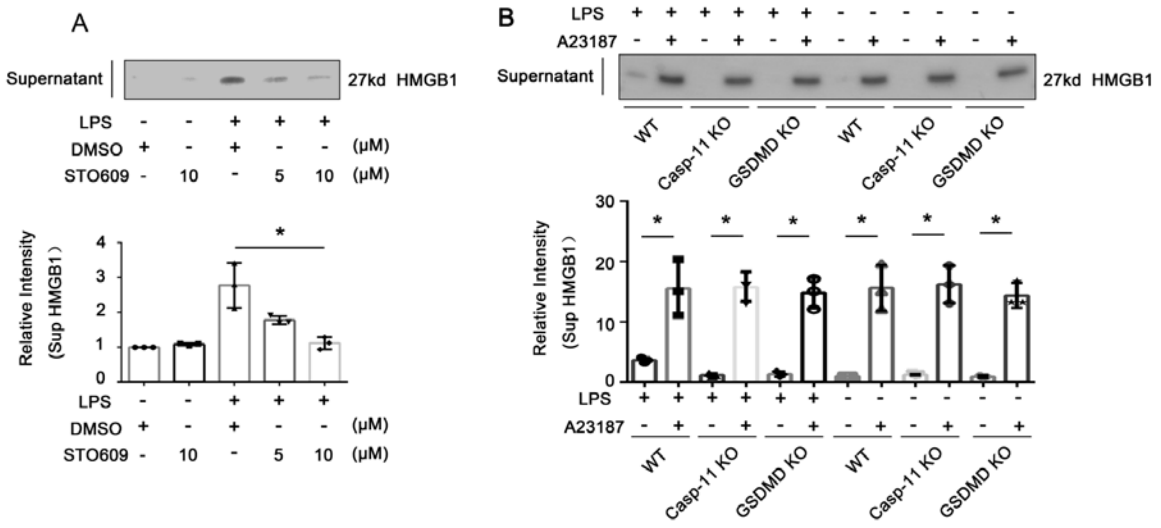

Supplemental Fig 4. STO-609 and A23187 effect on HMGB1 release from hepatocytes

A: WT hepatocytes were treated with STO-609 as the indicated concentration for 1h and then challenged with LPS (1ug/ml) for 24h. The Immuno-blots image shows one representative image for HMGB1 in the supernatant. Quantification charts show mean ± SEM of HMGB1 in the supernatant of three individual experiments. *P<0.05.

B: WT hepatocytes were treated with or without A23187 (5uM) for 1h and then challenged with or without LPS (1ug/ml) for 24h. The Immuno-blots image shows one representative image for HMGB1 in the supernatant. Quantification charts show mean ± SEM of HMGB1 in the supernatant of three individual experiments. *P<0.05.

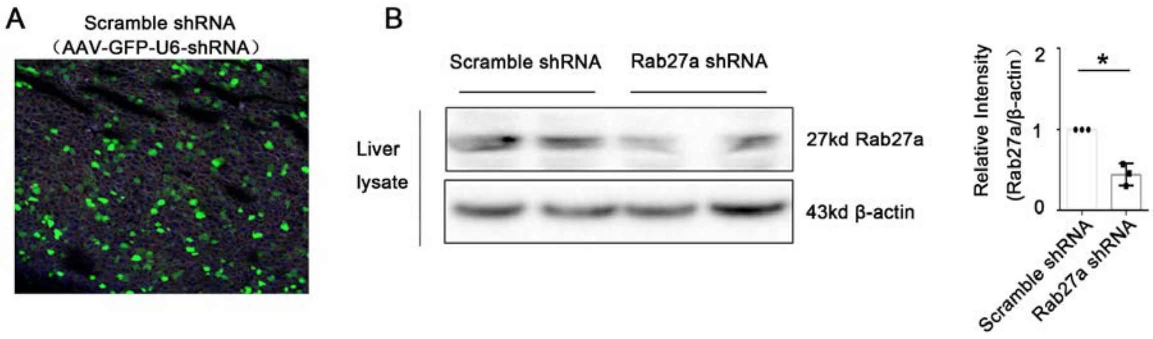

Supplemental Fig 5: Rab27a is successfully knockdown.

A: WT mice were injected into the penile vein with scramble shRNA for 48h. Liver section was observed by the confocal microscope.

B: WT mice were injected into the penile vein with scramble shRNA or Rab27a shRNA for 48h. Immuno-blots for Rab27a and β-actin in the liver tissue lysate.

The Immuno-blots image shows one representative image for Rab27a and β-actin in the liver lysate. Quantification charts show mean ± SEM of relative Rab27a intensity of three individual experiments. *$P<0.05$.

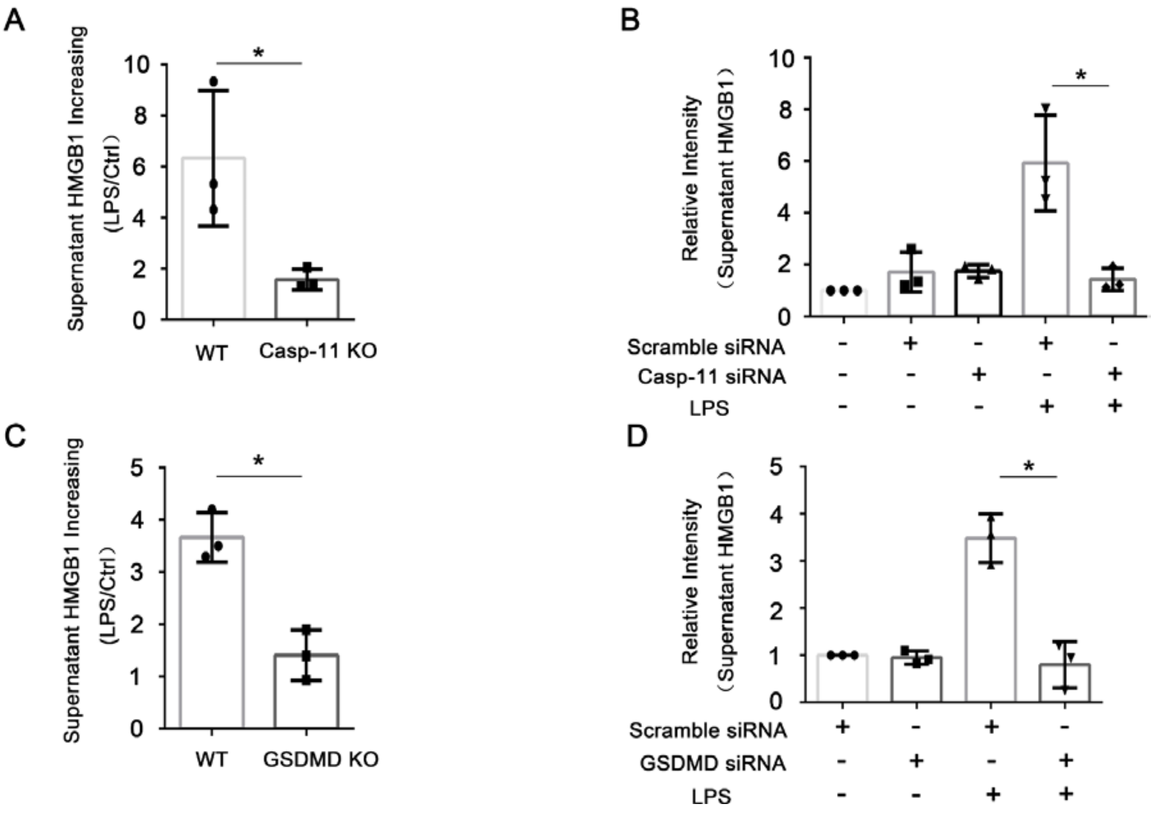

Supplemental Fig 6: Quantification of Immunoblots in Fig1

A, C: Immuno-blots image quantification charts show mean ± SEM of supernatant HMGB1 increasing of WT, caspase-11$^{-/-}$ (Caspase-11 KO) and GsdmD$^{-/-}$ (GsdmD KO) hepatocytes at 24h after LPS (1ug/ml) of three individual experiments. *P<0.05.

B, D: Hepatocytes pretreated with siRNA to knock down caspase-11 or GSDMD prior to LPS treatment for 24h. Immuno-blots image quantification charts show mean ± SEM of relative HMGB1 intensity in the supernatant of three individual experiments. *P<0.05.

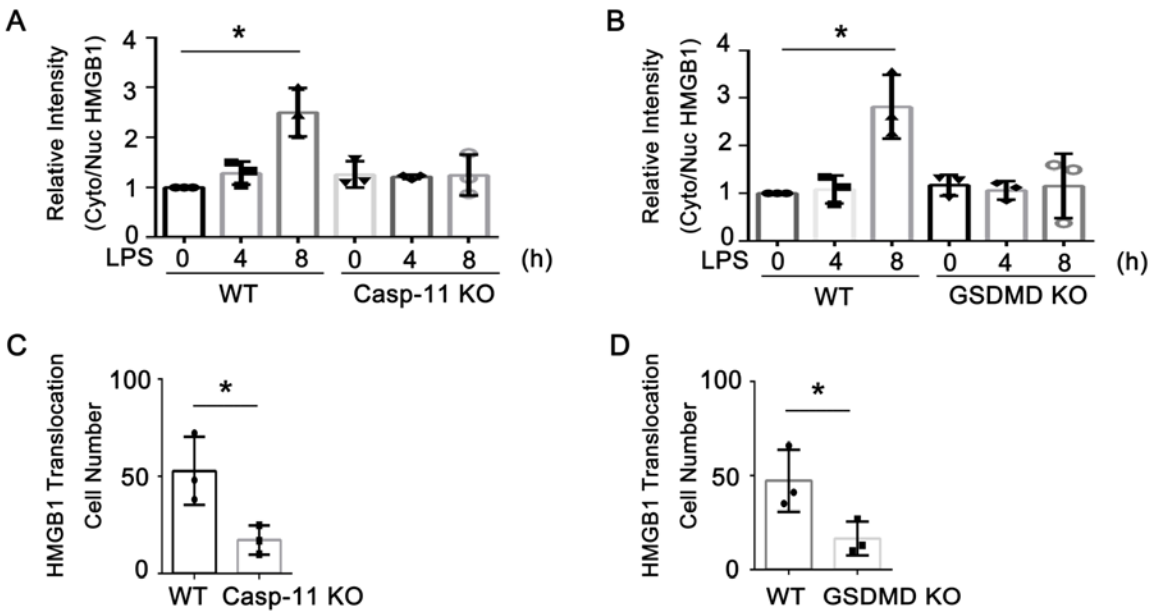

Supplemental Fig 7: Quantification of Western blot in Fig3

A，B: Immuno-blots image quantification charts show mean ± SEM of cytoplasmic (Cyto) HMGB1/ nucleus (Nuc) HMGB1 of WT, caspase-11-/- (Casp-11 KO) or GsdmD-/- (GSDMD KO) hepatocytes of three individual experiments. Cells were treated with LPS (1ug/ml) for time as indicated. *P<0.05.

C, D: Immunofluorescence quantification charts show mean ± SEM of HMGB1 translocation positive cells of WT, caspase-11$^{-/-}$ or GsdmD $^{-/-}$ hepatocytes treated with LPS (1ug/ml) for 8 hours of three individual experiments. HMGB1 translocation positive cells were evaluated and counted by two experienced individuals. *P<0.05.

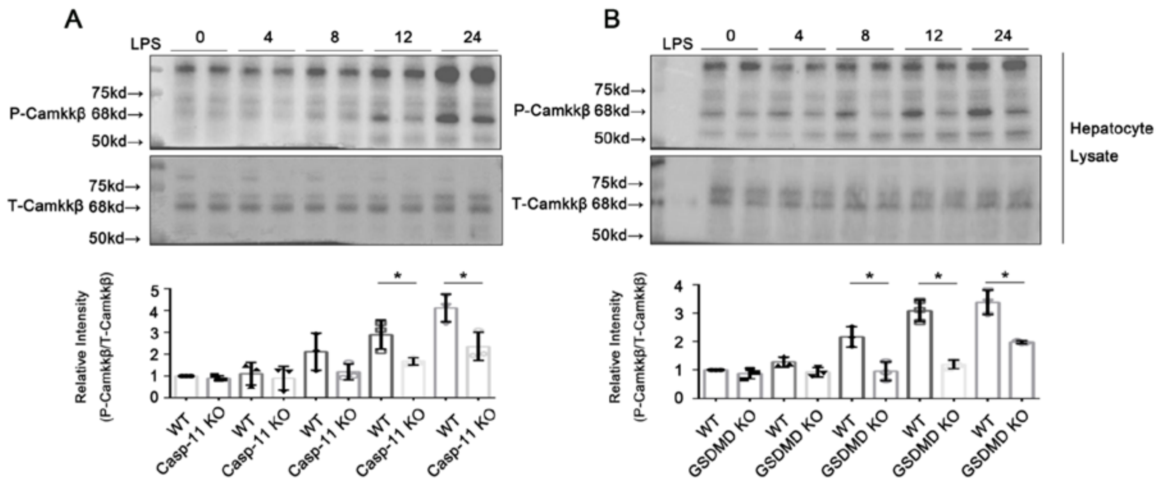

Supplemental Fig 8: Quantification of Western blot in Fig4

A, B: Immuno-blots image quantification charts show mean ± SEM of phospho-camkkβ (P-camkkβ) / total-camkkβ(T-camkkβ) in whole cell lysates of WT, caspase-11-/- (Casp-11 KO) or GsdmD-/- (GSDMD KO) hepatocytes of three individual experiments. Cells were treated with LPS (1ug/ml) for indicated times. *P<0.05.

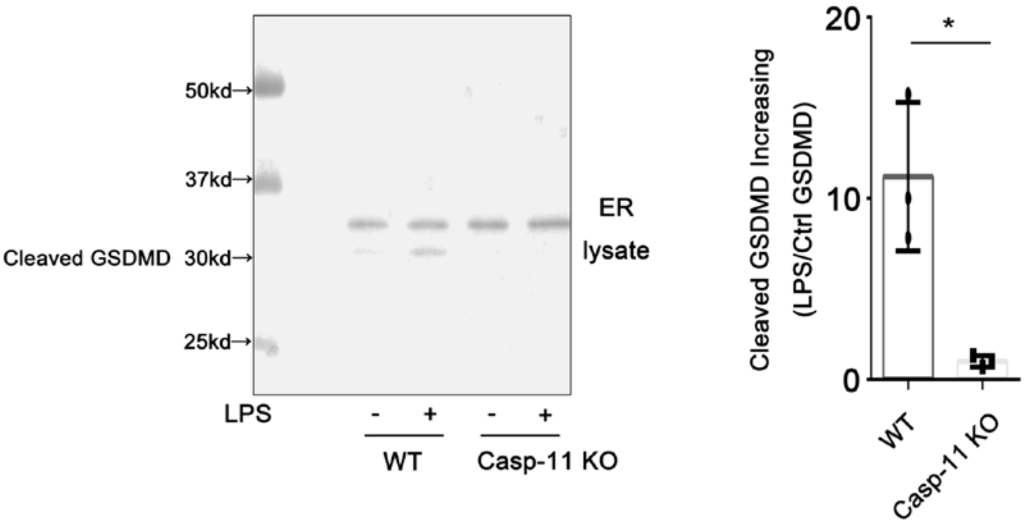

Supplemental Fig 9: Quantification of Western blot in Fig5

The Immuno-blots image shows one representative image for cleaved GSDMD in the endoplasmic reticulum (ER) lysate. Immuno-blots image quantification charts show mean ± SEM of cleaved GSDMD increasing in ER lysate of WT and caspase-11-/- (Casp-11 KO) hepatocytes of three individual experiments. Cells were treated with LPS (1ug/ml) for 8 hours. *P<0.05.

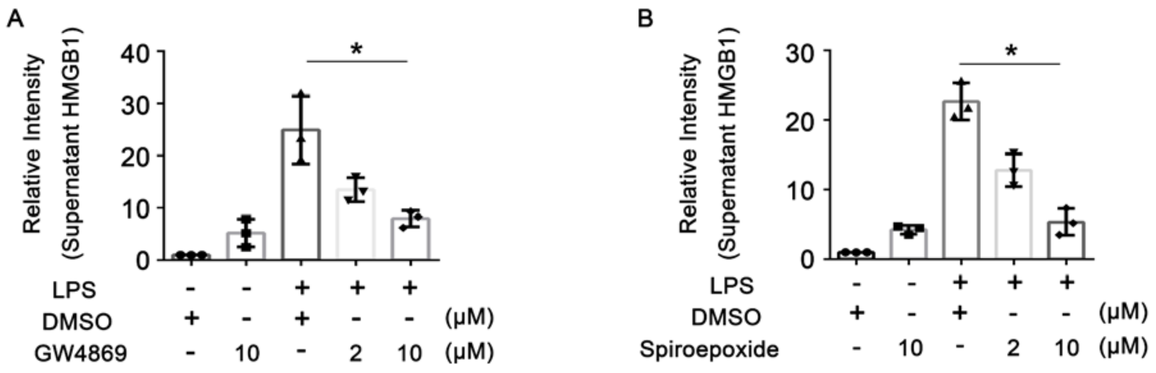

Supplemental Fig 10: Quantification of Western blot in Fig6

A, B: Immuno-blots image quantification charts show mean ± SEM of HMGB1 in the supernatant of WT hepatocytes of three individual experiments. Cells were treated with GW4869 or spiroepoxide for 2h, then challenged with LPS (1ug/ml) for 24h. *$P<0.05$.

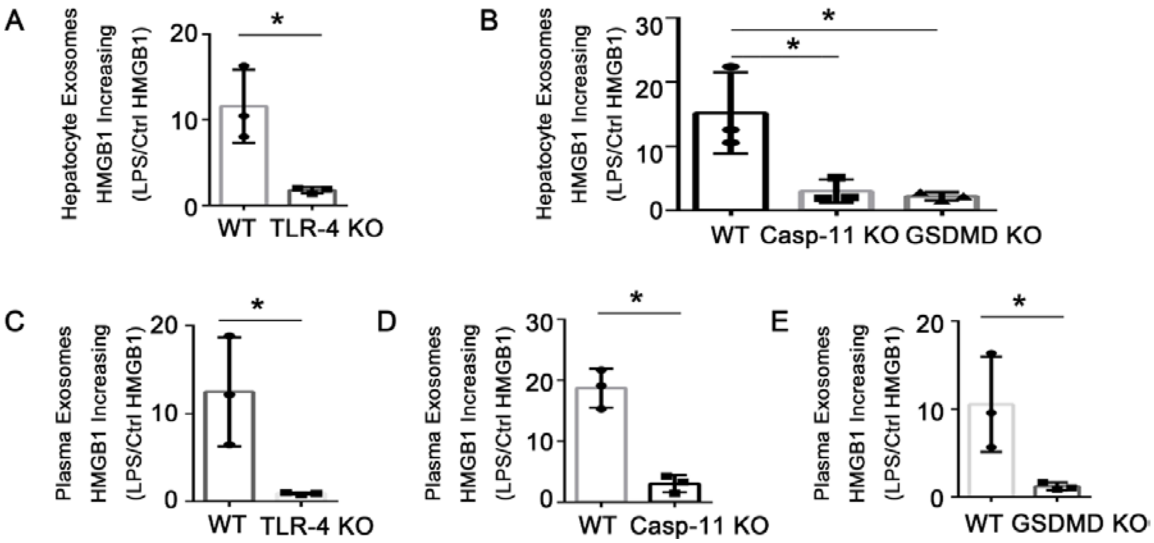

Supplemental Fig 11: Quantification of Western blot in Fig7

A, B: Immuno-blots image quantification charts show mean ± SEM of HMGB1 increasing in exosomes of WT, $TLR4^{-/-}$ (TLR4 KO), caspase-$11^{-/-}$ (Casp-11 KO) or $GsdmD^{-/-}$ (GSDMD KO) hepatocytes of three individual experiments. Cells were treated with LPS (1ug/ml) for 24h. *P<0.05.

C, D, E: Immuno-blots image quantification charts show mean ± SEM of HMGB1 increasing in plasma exosomes of WT, $TLR4^{-/-}$ (TLR4 KO), caspase-$11^{-/-}$ (Casp-11 KO) or $GsdmD^{-/-}$ (GSDMD KO) mice of three individual experiments. Mice were treated with LPS (1ug/ml) for 4h. *P<0.05.

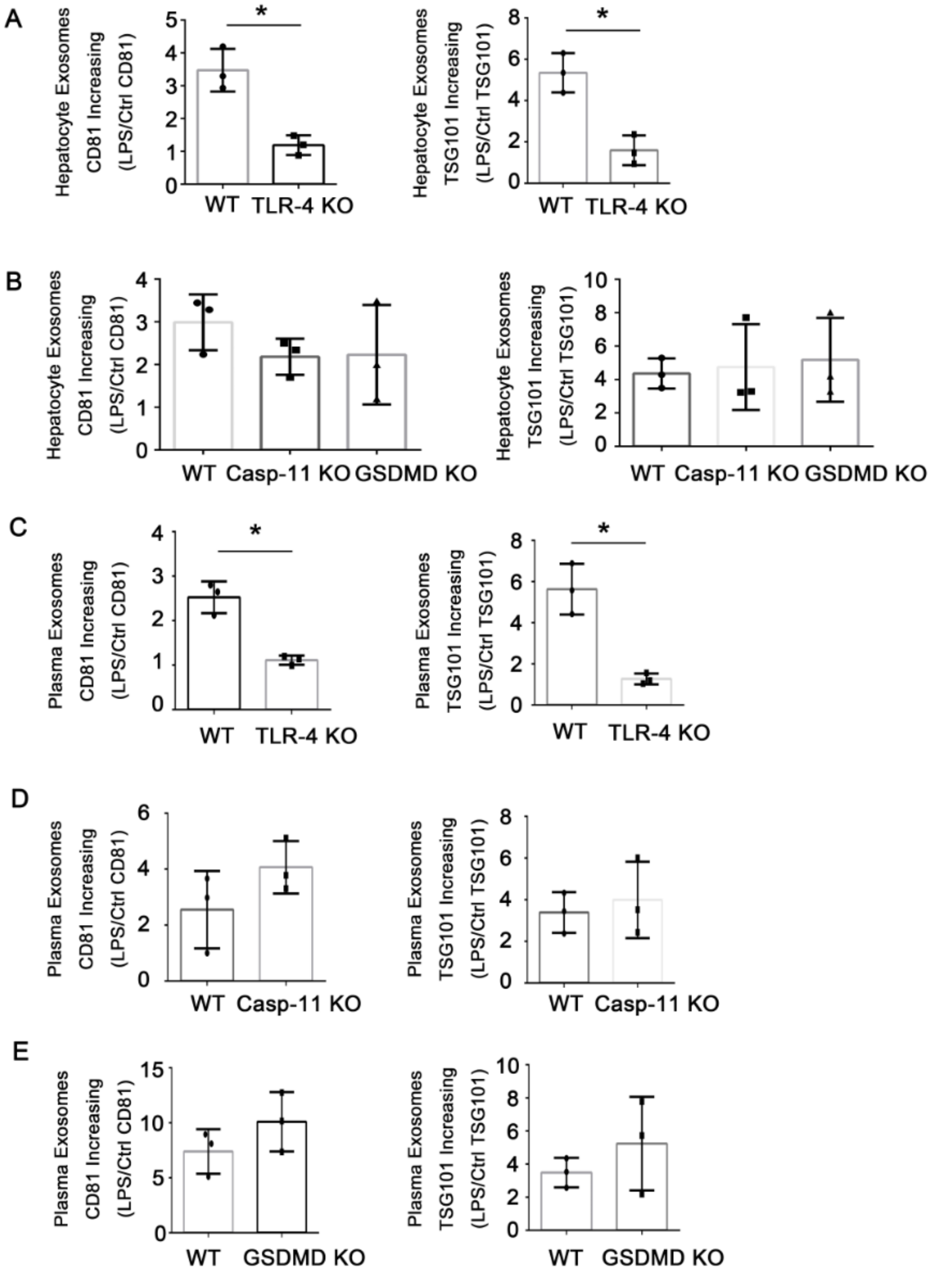

Supplemental Fig 12: Quantification of Western blot in Fig8

A, B: Immuno-blots image quantification charts show mean ± SEM of CD81 and TSG101 increasing in exosomes of WT, TLR4$^{-/-}$ (TLR4 KO), caspase-11$^{-/-}$ (Casp-11 KO) or GsdmD$^{-/-}$ (GSDMD KO) hepatocytes of three individual experiments. Cells were treated with LPS (1ug/ml) for 24h. *P<0.05.

C, D, E: Immuno-blots image quantification charts show mean ± SEM of CD81 and TSG101 increasing in plasma exosomes of WT, TLR4$^{-/-}$ (TLR4 KO), caspase-11$^{-/-}$ (Casp-11 KO) or GsdmD$^{-/-}$ (GSDMD KO) mice of three individual experiments. Mice were treated with LPS (1ug/ml) for 4h. *P<0.05.
